# Stage-associated differences in the serum *N*- and *O*-glycan profiles of patients with non-small cell lung cancer

**DOI:** 10.1186/s12014-019-9240-6

**Published:** 2019-05-10

**Authors:** Yiqian Liang, Peng Han, Ting Wang, Hui Ren, Lei Gao, Puyu Shi, Shuo Zhang, Aimin Yang, Zheng Li, Mingwei Chen

**Affiliations:** 1 Department of Respiratory and Critical Care Medicine, The First Affiliated Hospital of Xi’an Jiaotong University, Xi’an, 710061 China; 2 Department of Nuclear Medicine, The First Affiliated Hospital of Xi’an Jiaotong University, Xi’an, 710061 China; 3 Laboratory for Functional Glycomics, College of Life Sciences, Northwest University, Xi’an, 710069 China; 4 Department of Otolaryngology-Head and Neck Surgery, The First Affiliated Hospital of Xi’an Jiaotong University, Xi’an, 710061 China; 5 Department of Respiratory Medicine, Xi’an No.4 Hospital, Xi’an, 710004 China; 6 Department of Endocrinology, The Second Affiliated Hospital of Xi’an Jiaotong University, Xi’an, 710004 China; 7 Department of Radiology, The First Affiliated Hospital of Xi’an Jiaotong University, Xi’an, 710061 China

**Keywords:** Non-small cell lung cancer, Protein glycosylation, Serum biomarkers, Lectin microarray, Glycomics

## Abstract

**Background:**

Lung cancer is the leading cause of cancer death in China and around the world. Early detection is key to improving the survival rate of non-small cell lung cancer (NSCLC). Alteration in glycosylation has been observed in cancers, and glycans can be a source for the development of new biomarkers for NSCLC.

**Methods:**

In this glycan biomarker discovery study, we measured serum *N*- and *O*-glycan profiles in NSCLC patients with different stages and healthy controls by performing lectin microarray analysis. The alterations of serum glycopatterns were compared between NSCLC patients and controls, and the stage-related changes in serum glycosylation were evaluated.

**Results:**

There were 18 lectins (e.g., AAL, Jacalin, GSL-I and DBA) to give significantly alterations of serum glycopatterns in lung adenocarcinoma compared with control group. Meanwhile, 16 lectins (e.g., Jacalin, HHL, and PHA-E+L) exhibited significantly alterations of serum glycopatterns in squamous cell carcinoma (SCC) compared with control group. Importantly, most of the lectins showing altered signals exhibited significantly increased or decreased NFIs in patients with early stage adenocarcinoma and SCC.

**Conclusions:**

The serum glycan profiles were significantly different between NSCLC and healthy control, and most of the glycosylation changes had occurred at early stage. Further evaluation is needed to examine the diagnostic value of the glycan markers identified in this study.

**Electronic supplementary material:**

The online version of this article (10.1186/s12014-019-9240-6) contains supplementary material, which is available to authorized users.

## Background

In China, lung cancer is the most common incident cancer and the leading cause of cancer-related death [1]. According to the Global Burden of Disease Study 2016 (GBD2016), lung cancer had been identified as the fourth most frequent cause of death, and the death rate of lung cancer increased by 24.4% from 2005 to 2016 in China [2]. Non-small cell lung cancer (NSCLC), which accounts for 85% of all lung cancer cases, is divided into 3 major pathologic subtypes: adenocarcinoma, squamous cell carcinoma (SCC) and large cell carcinoma. Early diagnosis is critical because the chance of survival is greater in early stage (Stage I and II) NSCLC [3]. It is estimated that 36–73% of patients will survive longer than 5 years if the cancer is detected early [4]. At late stage (Stage III and IV), however, less than 15% will survive longer than 5 years.

Low-dose computed tomography (LDCT) has been suggested as a screening tool for early diagnosis of lung cancer [5], however its clinical utility is limited by its limited ability of differentiating benign pulmonary nodules from lung cancer. A systematic review of randomized clinical trials indicated that the average nodule detection rate of LDCT was 24.2%, with 96.4% of nodules being benign [6]. The high false positive rate of LDCT scan results in multiple screening rounds and unnecessary radiation exposure. The development of non-invasive complementary biomarkers could be a useful strategy to decrease false positive rate of CT scans and the overdiagnosis rate. A number of serum-based biomarkers have been identified and are being used clinically for lung cancer detection. Currently, carcinoembryonic antigen (CEA) is the most common clinical serum marker for lung adenocarcinoma. Cytokeratin 19 (CYFRA21-1) and squamous cell carcinoma antigen (SCCA) are commonly used as SCC markers. However, because of their low sensitivity, these markers described above have not been generally recommended as a tool for the early detection of lung cancer. During the early stages of NSCLC, the sensitivity of these markers is less than 25% [7]. Therefore, it is necessary to investigate novel serum biomarkers for lung cancer detection.

Aberrant glycosylation is observed in many types of cancers, and glycan-based biomarker discovery has advanced significantly recently [8–10]. Protein glycosylation involves addition of an oligosaccharide to either a nitrogen atom (*N*-linked glycosylation) or an oxygen atom (*O*-linked glycosylation) of the polypeptide chain. Glycosylation is the most common post-translation modification of secreted proteins, and alterations in cellular pathways can affect glycan structures [11]. The cancer-associated changes in glycosylation, including sialylation, fucosylation, *O*-glycan truncation, and *N*- and *O*-linked glycan branching, have been reported in the occurrence and progression of different cancers [12]. Aberrant glycosylation changes in the serum glycome could provide promising biomarkers for NSCLC.

In this glycan biomarker exploratory study, changes in serum glycan profiles was investigated in patients with NSCLC. We analyzed serum *N*- and *O*-glycan profiles in patients with NSCLC and healthy controls using lectin microarrays, a high-throughput glycome technique that we have proven to be useful in the given complex biological sample [13, 14]. In addition, glycan profiles of serum proteins from NSCLC patients with various stages were also compared.

## Methods

### Subject recruitment and sample collection

Serum samples were obtained from 62 newly diagnosed cases of primary NSCLC, including 28 adenocarcinoma and 34 SCC patients, at the First Affiliated Hospital of Xi’an Jiaotong University. The NSCLC cases should meet the following inclusion criteria: (1) newly diagnosed and confirmed histologically cases of primary NSCLC, (2) the patient had not received any treatment for NSCLC, (3) the patient had complete demographic and related clinical data. The study excluded the patients who did not meet the inclusion criteria and/or were diagnosed for any other primary cancer and lung cancer as their secondary primary cancer. Patients with NSCLC were confirmed by computed tomography guided fine needle aspiration cytology (FNAC) or fibre optic bronchoscopy (FOB) guided biopsy. Healthy control individuals were recruited from the health checkup center in the same hospital, and met the following inclusion criteria: (1) no clinical or radiological evidence of malignancy or other acute or chronic pathology, (2) the individual had complete demographic and related clinical data. Their cancer-free status was confirmed by testing for plasma levels of tumor biomarkers, including CEA, NSE, CYFRA21-1, SCCA, ProGRP, AFP, ferritin, PSA, CA-125, CA 15-3, CA 19-9 and CA 72-4, as well as computed tomography examination. Additionally, the subjects were chosen at the same or similar age stage. The samples of adenocarcinoma and control groups are gender-matched. Since SCC is rare in women compared with men in China, we only collected 1 female patient with SCC during the serum sample recruitment. Serum samples were kept at an − 80 °C freezer until processing.

### Serum protein labelling

We segregated adenocarcinoma and SCC patients using their disease stages based on the American Joint Committee on Cancer staging system. Among the 28 adenocarcinoma samples used for the detection of serum protein glycosylation, 8 were at Stage I/II, 8 at Stage III and 12 at Stage IV. Of the 34 SCC samples, 9 were at Stage I/II, 12 at Stage III and 13 at Stage IV. To normalize the differences between subjects and to tolerate individual variation, 100 μL of each sample from Stage I/II adenocarcinoma, Stage III adenocarcinoma, Stage IV adenocarcinoma, Stage I/II SCC, Stage III SCC, Stage IV SCC and healthy controls was, respectively, pooled. The pooled serum proteins were labelled with Cy3 fluorescence dye (GE Healthcare, Buckinghamshire, UK), and then labelled proteins were separated from the excess free dye by Sephadex G-25 columns (GE Healthcare) according to the manufacturer’s instructions. The Cy3-labeled serum proteins were quantified and stored at − 20 °C in the dark until use.

### Lectin microarrays and data analysis

A lectin microarray was produced using 37 lectins (Vector Laboratories, Sigma-Aldrich, and Calbiochem) with different specificities covering *N*- and *O*-linked glycans as previously described [13, 15]. The sugar-binding specificities of lectins and the layout of the lectin microarrays are summarized in Additional files, Additional file 1: Fig. S1 and Additional file 2: Table S1. Each lectin was printed in triplicate per block with triplicate blocks on one slide. BSA and BSA-conjugated with Cy3 were used to validate the feasibility of the lectin microarray. After immobilization, the slides were blocked with blocking buffer (2%, w/v, BSA in PBS, pH 7.4) for 1 h and rinsed twice with PBS. Next, 4 μg of Cy3-labeled serum proteins was diluted to 700 μL with hybridization buffer (2%, w/v, BSA, 500 mM glycine and 0.1%, v/v, Tween-20 in PBS, pH 7.4), and the resultant Cy3-labeled protein solution was applied to the lectin microarrays. After incubation and washing, the slides were scanned using a Genepix 4000B confocal scanner set at 70% photomultiplier tube and 100% laser power (Axon Instruments, Union City, CA).

The acquired images were analyzed at 532 nm by Genepix 3.0 software (Axon Instruments). The average background was subtracted, and values less than the average background ± 2 standard deviations (SD) were removed from each data point. The median of the effective values of each lectin was normalized by the sum of the medians of all effective data points for each lectin in one block. The pooled serum of each group was observed by 3 repeated microarrays and the normalized medians of each lectin from 9 repeated blocks were averaged and its SD was calculated.

### Statistical analysis

All statistical analysis was done with the Statistical Package for the Social Sciences (SPSS) version 20.0 (SPSS Inc., Chicago, IL). Differences between two arbitrary datasets were evaluated using one-way analysis of variance (ANOVA) or Student’s *t* tests. Hierarchical clustering analysis (HCA) was performed by Expander 7.1 (http://acgt.cs.tau.ac.il/expander/). *P* values of < 0.05 were considered statistically significant.

## Results

### Serum glycopatterns in patients with NSCLC and healthy controls

**Fig. 1:**
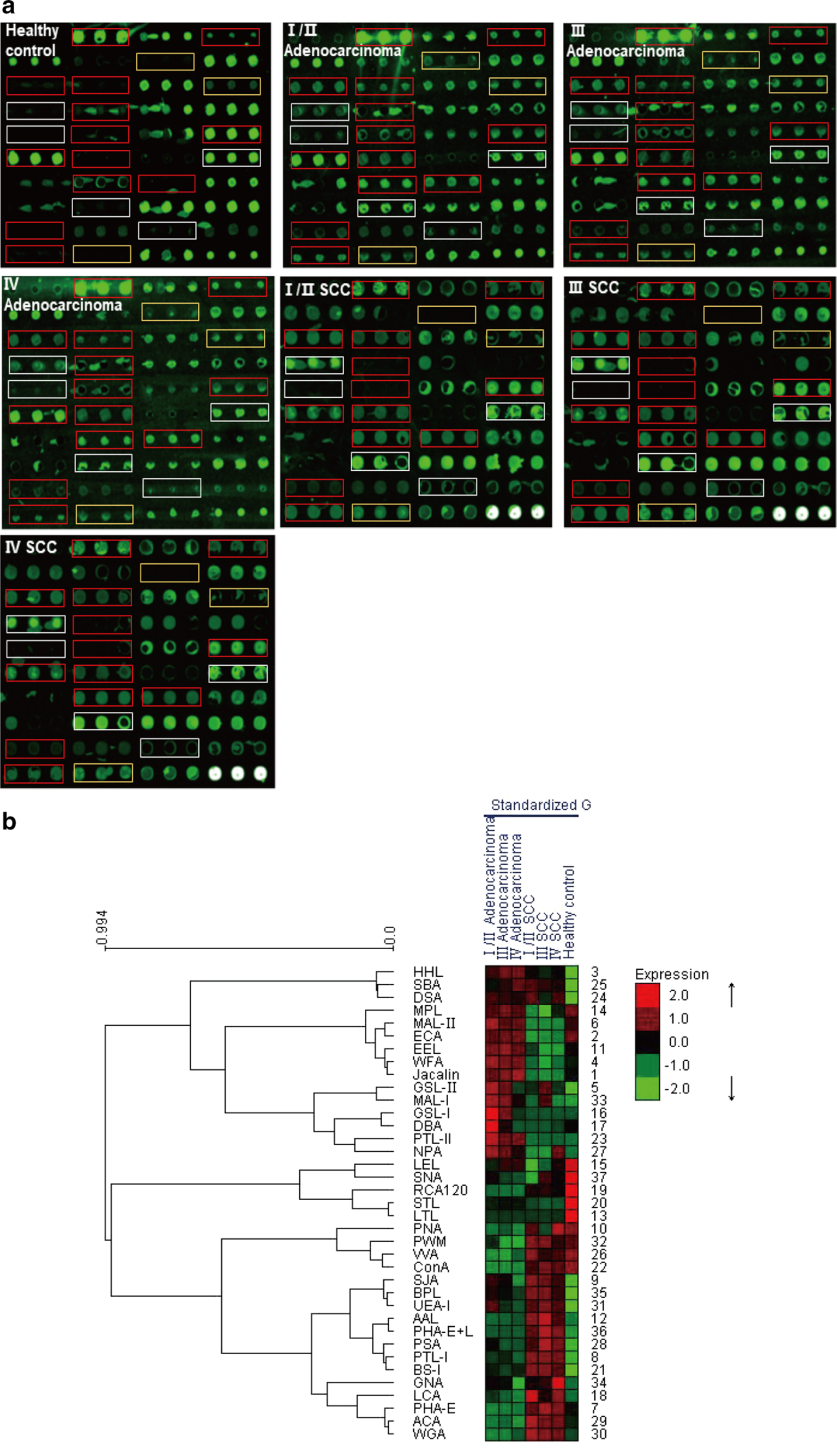
The glycopatterns of serum glycoproteins from NSCLC patients and healthy controls using the lectin microarrays. **a** The glycopatterns of Cy3-labeled serum proteins from Stage I/II adenocarcinoma, Stage III adenocarcinoma, Stage IV adenocarcinoma, Stage I/II SCC, Stage III SCC, Stage IV SCC and healthy controls bound to the lectin microarrays. A block of the slide with 3 replicates of 37 lectins was shown. Lectins showing the difference between adenocarcinoma and control were marked with white frames; lectins showing the difference between SCC and control were marked with yellow frames; and compared with control group, lectins showing the difference in both adenocarcinoma and SCC groups were marked with red frames. **b** Heat map and hierarchical clustering analysis of the normalized data of 37 lectins in the 7 groups. The groups were listed in columns, and the lectins were listed in rows. The color and intensity of each square indicated lectin signals relative to other groups in the row. Red, high; green, low; black, medium

The characteristics of study participants are summarized in Table 1. The glycopatterns of Cy3-labeled serum proteins from Stage I/II adenocarcinoma, Stage III adenocarcinoma, Stage IV adenocarcinoma, Stage I/II SCC, Stage III SCC, Stage IV SCC and control subjects bound to the lectin microarrays are shown in Fig. 1a. Their normalized fluorescent intensities (NFIs) for each lectin are summarized in Additional file, Additional file 3: Table S2. The normalized data of the 7 groups were imported into Expander 7.1 to perform a hierarchical clustering analysis (Fig. 1b). Lectin signal patterns were classified into 3 categories to evaluate whether the glycan profiles of serum glycoproteins were altered in NSCLC: (1) those showing significant increases in NFIs (fold change > 1.5, *P* < 0.05), (2) those showing significant decreases in NFIs (fold change < 0.67, *P* < 0.05), and (3) those showing almost even level in NFIs (fold change range from 0.67 to 1.5, no significant difference) [14, 15]. Those lectins showing the difference of glycan expression between NSCLC and healthy control were marked with frames in Fig. 1a.

**Table 1:** Characteristics of the NSCLC patients and healthy controls

Characteristics	Adenocarcinoma (stage)			SCC (stage)			Healthy control
	I/II	III	IV	I/II	III	IV	
Age							
Years, mean ± SD	63 ± 5.2	56 ± 9.5	57 ± 10.3	64 ± 7.8	61 ± 9.0	61 ± 8.6	69 ± 6.6
Gender							
Male	3	4	7	9	12	12	6
Female	5	4	5	0	0	1	6
Smoking status							
Ever	4	4	7	8	12	12	7
Never	4	4	5	1	0	1	5

SCC, squamous cell carcinoma; SD, standard deviation

### Alterations of serum glycopatterns between adenocarcinoma patients and control individuals

Eighteen lectins that showed significant differences in glycan expression of serum glycoproteins between the adenocarcinoma and healthy control groups are shown in Table 2. Among the 14 lectins exhibiting increased NFIs in adenocarcinoma groups compared to control group, 11 lectins, e.g. the Fucα1-6GlcNAc (core fucose) binder AAL, the GlcNAc and αGal binder GSL-I, and the terminal GalNAc binder SBA, showed significantly increased NFIs in Stage I/II, Stage III and Stage IV adenocarcinoma groups. Compared with control group, the Galβ1-3GalNAcα-Ser/Thr (T antigen) binder Jacalin and the Fucα1-2Galβ1-4GlcNAc binder UEA-I showed increased NFIs in Stage III and Stage IV adenocarcinoma, while the GalNAcα-Ser/Thr (Tn antigen) and GalNAcα1-3Gal binder DBA only showed increased NFIs in Stage I/II adenocarcinoma. Moreover, compared with control group, the Fucα1-2Galβ1-4GlcNAc, Fucα1-3(Galβ1-4)GlcNAc binder LTL, the β-Gal, Galβ-1,4GlcNAc (type II) and Galβ1-3GlcNAc (type I) binder RCA-120, the (GlcNAc)_n_ binder STL, the high-Mannose, Manα1-6(Manα1-3)Man binder ConA showed significantly decreased NFIs in adenocarcinoma groups. The results indicated that the significant differences in the signals of 12 lectins showing increased NFIs and 4 lectins showing decreased NFIs had occurred in patients with early stage lung adenocarcinoma.

**Table 2:** Differences in the glycopattern of serum proteins between adenocarcinoma groups and healthy control by the lectin microarray analysis based on data of 18 lectins giving significant differences

Lectin	Specificity	Fold change		
		I/II Adenocarcinoma/healthy	III Adenocarcinoma/healthy	IV Adenocarcinoma/healthy
Jacalin	Galβ1-3GalNAcα-Ser/Thr(T), sialyl-T(ST)	1.68	1.86***	2.11**
HHL	High-Mannose, Manα1-3Man, Manα1-6Man, Man5-GlcNAc2-Asn	2.85**	3.16*	3.13*
PTL-I	GalNAc, GalNAcα-1, 3Gal, GalNAcα-1, 3Galβ-1,3/4Glc	3.49**	3.85**	2.81*
SJA	Terminal in GalNAc and Gal	2.98**	2.73**	1.55
AAL	Fucα1-6GlcNAc(core fucose), Fucα1-3 (Galβ1-4) GlcNAc	2.09***	2.71**	3.57**
LTL	Fucα1-2Galβ1-4GlcNAc, Fucα1-3(Galβ1-4) GlcNAc	0***	0***	0**
GSL-I	αGalNAc,GalNAcα-Ser/Thr (Tn) and αGal	/**	/**	/***
DBA	GalNAcα-Ser/Thr(Tn) and GalNAcα1-3Gal	4.27*	–	–
RCA-120	β-Gal, Galβ-1, 4GlcNAc(type II), Galβ1-3GlcNAc (type I)	0.13**	0.10***	0.09**
STL	Trimers and tetramers of GlcNAc, core (GlcNAc) of *N*-glycan, oligosaccharide containing GlcNAc and MurNAc	0.43**	0.45**	0.44**
BS-I	αGal and αGalNAc	/***	/***	/**
ConA	High-Mannose, Manα1-6(Manα1-3)Man, terminal GlcNAc	0.35***	0.35***	0.36***
DSA	β-D-GlcNAc, (GlcNAcβ1-4)_n_, Galβ1-4GlcNAc	3.77***	3.62***	3.49***
SBA	Terminal GalNAc (especially GalNAcα1-3Gal)	11.75***	13.96***	16.31***
PSA	α-D-Man, Fucα-1, 6GlcNAc (core fucose), α-D-Glc	9.36**	5.92***	6.20**
UEA-I	Fucα1-2Galβ1-4GlcNAc	7.99	5.29***	4.46**
MAL-I	Galβ-1,4GlcNAc, Siaα2-3Gal, Galβ1-3GlcNAc, Siaα2-3	/**	/***	/***
BPL	Galβ1-3GalNAc, terminal GalNAc	/**	/*	/*

–, no significant difference; /, the denominator of the fold change was zero

*P < 0.05, **P < 0.01, ***P < 0.001

**Fig. 2:**
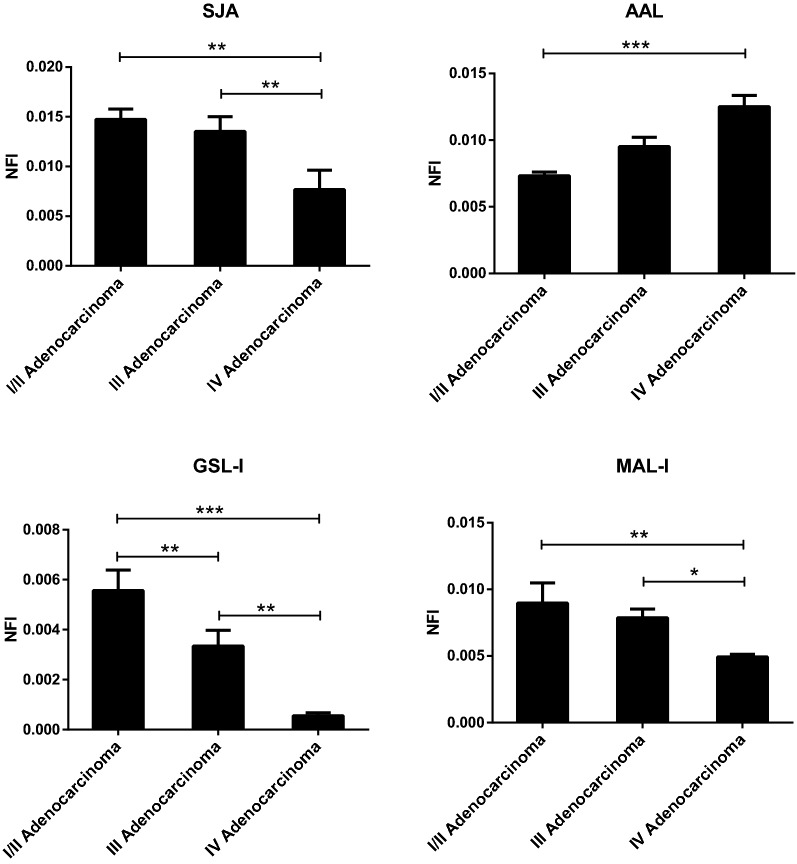
Lectins revealed significant differences among Stage I/II, Stage III, and Stage IV adenocarcinoma groups according to ANOVA. *P < 0.05, **P < 0.01, ***P < 0.001

Further statistical analysis was performed to evaluate the stage-related changes in NFIs of the 18 lectins showing altered signals in adenocarcinoma patients. The NFIs of each lectin from 3 stage groups were compared based upon fold changes and *P* value in pairs (Fig. 2). The NFIs of AAL had an increasing trend with stage in lung adenocarcinoma and was significantly higher in Stage IV adenocarcinoma than in Stage I/II adenocarcinoma. The NFIs of SJA, GSL-I and MAL-I were increased in lung adenocarcinoma compared to healthy control, but they had a decreasing trend with stage in adenocarcinoma patients. The NFIs of SJA and MAL-I showed significantly lower NFIs in Stage IV adenocarcinoma compared with those in Stage I/II and Stage III adenocarcinoma. The NFIs of GSL-I showed significant differences between each pair of the 3 stage groups.

### Alterations of serum glycopatterns between patients with SCC and control individuals

The NFIs of 16 lectins were significantly different between SSC and control groups. As shown in Table 3, there were 9 lectins to show stronger signal with the fold change greater than 1.5 in SCC groups compared with control group, for example, the high-Mannose, Manα1-3Man, and Manα1-6Man binder HHL, the bisecting GlcNAc and biantennary *N*-glycans binder PHA-E+L, etc. Meanwhile, 7 lectins, including the T antigen binder Jacalin, the Galα1-3(Fucα1-2)Gal binder EEL, the Fucα1-2Galβ1-4GlcNAc and Fucα1-3(Galβ1-4)GlcNAc binder LTL, and the GalNAcα-Ser/Thr and GalNAcα1-3Gal binder DBA, showed weak signal with the fold change lower than 0.67 in SSC groups compared with control group. All the lectins showing different signals in SCC groups, except EEL, exhibited significantly increased or decreased NFIs in patients with early stage SCC compared with healthy controls. In addition, further analysis on stage-related alterations indicated that the NFIs of these lectins were not significantly different among Stage I/II, Stage III, and Stage IV SCC groups.

**Table 3:** Differences in serum glycopatterns between SCC groups and healthy control by the lectin microarray analysis based on data of 16 lectins giving significant differences

Lectin	Specificity	Fold change		
		I/II SCC/healthy	III SCC/healthy	IV SCC/healthy
Jacalin	Galβ1-3GalNAcα-Ser/Thr(T), sialyl-T(ST)	0.30***	0.17***	0.23***
HHL	High-Mannose, Manα1-3Man, Manα1-6Man, Man5-GlcNAc2-Asn	2.59*	2.32*	2.52*
MAL-II	Siaα2-3Galβ1-4Glc(NAc)/Glc, SLe^x^	0**	0**	0**
PTL-I	GalNAc, GalNAcα-1, 3Gal, GalNAcα-1, 3Galβ-1,3/4Glc	6.63**	6.61***	6.36**
SJA	Terminal in GalNAc and Gal	3.61**	3.37**	3.01**
EEL	Galα1-3(Fucα1-2)Gal	0.66	0.11***	0.14***
LTL	Fucα1-2Galβ1-4GlcNAc, Fucα1-3 (Galβ1-4) GlcNAc	0**	0**	0**
DBA	GalNAcα-Ser/Thr(Tn) and GalNAcα1-3Gal	0*	0^*^	0*
RCA-120	β-Gal, Galβ-1, 4GlcNAc (type II), Galβ1-3GlcNAc (type I)	0.44**	0.46***	0.39**
STL	Trimers and tetramers of GlcNAc, core (GlcNAc) of *N*-glycan, oligosaccharide containing GlcNAc and MurNAc	0.35**	0.31**	0.33**
BS-I	αGal and αGalNAc	/**	/***	/**
DSA	β-D-GlcNA, (GlcNAcβ1-4)_n_, Galβ1-4GlcNAc	4.02***	2.94	3.77***
SBA	Terminal GalNAc (especially GalNAcα1-3Gal)	15.57***	14.21***	13.32***
UEA-I	Fucoseα1-2Galβ1-4GlcNAc	8.00*	8.81***	7.86***
BPL	Galβ1-3GalNAc, terminal GalNAc	/***	/***	/**
PHA-E+L	Bisecting GlcNAc, biantennary *N*-glycans and tetraantennary complex-type *N*-glycan	8.80***	11.52**	9.86**

*SCC* squamous cell carcinoma

/, the denominator of the fold change was zero

*P < 0.05, **P < 0.01, ***P < 0.001

## Discussion

Protein modifications, which occur as translational, post-translational, regulatory, and/or degradation products, play important roles in biological processes and increase the amount of useful cancer-related information obtained from the proteins [16]. Glycosylation is involved in various aspects of cancer development, including cell–cell interactions, cell adhesion, malignant transformation and metastasis. Driven by technology advancements in the field of analytical glycoscience, protein glycosylation has become increasingly important in cancer research and hold considerable promise to provide new candidate glycan markers for cancer. Aberrant glycosylation of serum proteins has been found in patients with various types of cancer, including breast, colon, ovarian, and pancreatic cancer [17, 18]. Therefore, glycan-based serological assays may be useful to detect serum biomarkers for cancer. Kyselova et al. performed a MALDI mass spectrometry (MS)-based glycomic profile analysis, and found increases in sialylation and fucosylation of serum *N*-glycans in breast cancer appeared to be indicative of cancer progression [19]. In another study, Akimoto et al. found that 9 glycans were highly expressed in the sera of patients with invasive intraductal papillary mucinous neoplasms (IPMNs), and fucosylated tri-antennary glycan could distinguish invasive IPMNs from non-invasive IPMNs [10]. In this study, a lectin microarray was used firstly to investigate the glycan profiles of serum proteins in NSCLC patients at different stages, and systematically compare the alterations of serum glycopatterns between NSCLC patients and healthy controls. There were 18 lectins (e.g., AAL, Jacalin, GSL-I and DBA) to give significantly alterations of serum glycopatterns in lung adenocarcinoma patients compared with healthy control. Meanwhile, compared to control group, 16 lectins (e.g., Jacalin, HHL, and PHA-E+L) exhibited significantly alterations of serum glycopatterns in SCC patients.

Although fucosylation is essential for normal biological functions, a change in fucosylation has been strongly implicated in tumor development and progression. In the present study, we found that the levels of core fucosylation identified by AAL was elevated in sera from lung adenocarcinoma patients. Importantly, since the serum levels of core fucosylation were significantly increased in Stage I/II adenocarcinoma compared with control group, the core fucosylation might be a potential diagnostic biomarker for early detection of lung adenocarcinoma. Core fucosylation is the addition of α1-6-linked fucose to the innermost GlcNAc residue of *N*-linked glycans, which is catalyzd by fucosyltransferase 8 (FUT8) [20]. The elevated core fucosylation has been also observed in sera of hepatocellular carcinoma (HCC), prostate and ovarian cancer patients [8, 21, 22]. In addition to the increase in core fucosylation, the transcription of FUT8 was enhanced in HCC and prostate cancer tissues [23, 24]. It should also be noted that a recent study by Mehta et al. observed a similar level of core fucosylation in HCC tissue compared to the adjacent nontumor tissue, after measuring increased expression level of FUT8 in HCC tissue compared to nontumor tissue, suggesting that there might be an abnormal secretion of core fucosylated proteins into the serum of HCC patients [25]. Two studies by Chen et al. and Honma et al. showed that the expression of FUT8 was up-regulated in NSCLC tissues, and it was significantly more prevalent in adenocarcinoma than in SCC [26, 27]. In line with these results mentioned above, the elevated level of core fucosylation was observed in the sera of adenocarcinoma patients, but not in SCC patients in this study. Moreover, according to the lectin microarray results, the serum levels of core fucosylation had an increasing trend with stage in adenocarcinoma. The increased serum level of core fucosylation may reflect the tumor burden and serve as a prognostic marker for lung adenocarcinoma.

In this study, we found that the signals of T/Tn antigen binders Jacalin, GSL-I and DBA was increased in the sera of patients with adenocarcinoma, while Jacalin and DBA showed decreased NFIs in the sera of SCC patients. Tn antigen is a common precursor of *O*-linked glycans. With the aid of T-synthase, Tn antigen can receive a galactose unit (from UDP-Gal) to form T antigen (Thomsen-Friedenreich antigen). It has been reported that Tn antigen was highly expressed in 10–90% of breast, colon, cervix, stomach, and prostate tumors, which indicated the relationship between Tn antigen and cancer [28]. T antigen was detected as a cancer-associated carbohydrate antigen, and involved in tumor cell adhesion and tissue invasion [29]. Consistent with our results, Lopez-Ferrer et al. found that T and Tn antigen were detected more frequently in lung adenocarcinomas than in lung squamous cell carcinomas by immumohistochemical staining [30]. In another study, the levels of T antigen and its respective binding site were significantly lower in the head and neck squamous cell carcinoma samples compared with the normal samples [31]. Given the differential expressions of T and Tn antigen in adenocarcinoma and SCC, these markers may have the potential to distinguish the histological subtypes of NSCLC. A recent study on breast cancer cell lines indicated that in the highly metastatic cell line MDA-MB435, enhanced level of T antigen was found, but the T antigen level was much lower in the non-metastatic cell line MDA-MB468 [28]. The present study also found that the serum level of T antigen was only elevated in the patients with Stage III and Stage IV adenocarcinoma compared with healthy controls. Hence, the expression of T antigen might be associated with enhanced metastatic potential and poor prognosis in caners, and its detection is probably useful in serologic diagnosis.

Previous studies have indicated that elevated high-mannose *N*-linked glycans were associated with various cancers including ovarian, breast and colorectal cancer [9, 32, 33]. De Leoz et al. reported an elevation of high-mannose glycans in the sera of patients with metastatic breast cancer compared with healthy controls [9]. In glycan biosynthesis, the high-mannose structures are synthesized early in the process. The mannose residues of the *N*-glycans are then cleaved sequentially and are replaced by *N*-acetylglucosamine and glucose residues to form complex and hybrid glycans. The elevated levels of secreted high-mannose glycans suggested that the incomplete maturation of *N*-glycosylation in the secretory pathway, which could result from higher protein secretion and/or lower abundance/activity of the glycosylation enzymes [34]. In this study, we found that the level of high-mannose glycans identified by HHL was significantly increased in the sera of SSC patients compared with control group. The increased high mannose structures could influence the function of glycoproteins in serum through altering the stability, adhesion and communication properties of the protein [9].

There are still some limitations in this study. Lectin microarray is a high-cost and time-consuming procedure and not suitable for large number of samples. Therefore, a sample pooling strategy was employed to overcome this drawback. However, the pooled samples are unable to provide individual information of glycan expression. The data of pooled samples cannot be used to perform receiver operating characteristic curve analysis and calculate the cut-off value, sensitivity and specificity of the differentially expressed glycopatterns for NSCLC detection. Moreover, we pooled serum samples with an equal volume, which can globally reveal the differences of glycan expression in serum glycoproteins between patients with NSCLC and controls. And meanwhile, this pooling method can keep the concentration differences of both glycoproteins and glycans in each serum sample better than pooling samples with an equal total amount of serum proteins. However, the total protein content of sera depends individually on the nutrient, body fluid alteration, age, and physical and disease conditions. If the total serum protein concentration was significantly lower than the normal level in some patients, which influenced low-abundance proteins, pooling equal volumes of samples might give rise to some biases. Future studies are required to detect these aberrant glycopatterns in individual serum samples to verify their diagnostic value (sensitivity and specificity) as potential biomarkers for NSCLC. We will also perform studies to determine the diagnostic values of these glycan biomarkers for distinguishing NSCLC from benign pulmonary diseases and other types of cancers in the future.

Although this study requires further testing and validation, it suggests that the glycan profiles of serum proteins are significantly different between NSCLC and healthy control. Analysis of serum glycans by lectin microarray represents a new paradigm in cancer biomarker studies, i.e. a focus on glycosylation modification of proteins rather than protein expression. Most of the glycosylation changes in the sera of NSCLC patients were observed at early stage of this disease. Further investigation should be performed to evaluate the early diagnostic value of these serum markers for NSCLC in the future.

## Additional files

**Additional file 1: Figure S1.** The layout of the lectin microarrays. Each lectin was spotted in triplicate per block, with triplicate blocks on one slide. Cy3-labeled BSA was spotted as a location marker and BSA as a negative control.

**Additional file 2: Table S1.** Sugar-binding specificities of the 37 lectins used in the lectin microarray.

**Additional file 3: Table S2.** The normalized fluorescent intensities (NFIs) of each lectin for the serum proteins from NSCLC groups and control group.

## Abbreviations

NSCLC: non-small cell lung cancer
SCC: squamous cell carcinoma
LDCT: low-dose computed tomography
CEA: carcinoembryonic antigen
SCCA: squamous cell carcinoma antigen
NSE: neuron specific enolase
ProGRP: pro-gastrin-releasing peptide
AFP: α-fetoprotein
PSA: prostate-specific antigen
SD: standard deviations
HCA: hierarchical clustering analysis
NFI: normalized fluorescent intensity
IPMN: intraductal papillary mucinous neoplasm
FUT8: fucosyltransferase 8
HCC: hepatocellular carcinoma
T antigen: Thomsen–Friedenreich antigen
