# Supplementary figures and images for: Stage-associated differences in the serum N- and O-glycan profiles of patients with non-small cell lung cancer
Source: Clin Proteomics. 2019 May 10;16:20. doi: 10.1186/s12014-019-9240-6 (PMC6509814; doi:10.1186/s12014-019-9240-6)

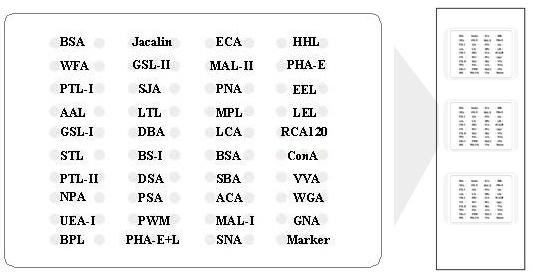

Supplement: Supplementary file 1 — Additional file 1: Figure S1. The layout of the lectin microarrays. Each lectin was spotted in triplicate per block, with triplicate blocks on one slide. Cy3-labeled BSA was spotted as a location marker and BSA as a negative control. [file 12014_2019_9240_MOESM1_ESM.jpg]
