# Supplementary material for: Stage-associated differences in the serum N- and O-glycan profiles of patients with non-small cell lung cancer
Source: Clin Proteomics. 2019 May 10;16:20. doi: 10.1186/s12014-019-9240-6 (PMC6509814; doi:10.1186/s12014-019-9240-6)
Supplement: Supplementary file 2 — Additional file 2: Table S1. Sugar-binding specificities of the 37 lectins used in the lectin microarray. [file 12014_2019_9240_MOESM2_ESM.pdf]

Table S1. Sugar-binding specificities of the lectins

Lectins

Artocapus integrifolia (Jacalin), Erythrina cristagalli (ECA), Hippeastrum Hybrid Lectin (HHL), Wisteria Floribunda Lectin (WFA), Griffonia (Bandeiraea) Simplicifolia Lectin II (GSL-II), Maackia Amurensis Lectin II (MAL-II), Phaseolus vulgaris Agglutinin(E) (PHA-E), Psophocarpus Tetragonolobus Lectin I (PTL-I), Sophora Japonica Agglutinin (SJA), Peanut Agglutinin (PNA), Euonymus Europaeus Lectin (EEL), Aleuria Aurantia Lectin (AAL), Lotus Tetragonolobus Lectin (LTL), Maclura Pomifera Lectin (MPL), Lycopersicon Esculentum (Tomato) Lectin (LEL), Griffonia (Bandeiraea) Simplicifolia Lectin I (GSL-I), Dolichos Biflorus Agglutinin (DBA), Lens Culinaris Agglutinin (LCA), Ricinus Communis Agglutinin I (RCA120), Solanum Tuberosum (Potato) Lectin (STL), Bandeiraea simplicifolia (BS-I), Canavalia ensiformis (ConA), Psophocarpus Tetragonolobus Lectin II (PTL-II), Datura stramonium (DSA), Soybean Agglutinin (SBA), Vicia Villosa Lectin (VVA), Narcissus Pseudonarcissus Lectin (NPL), Pisum Sativum Agglutinin (PSA), Amaranthus caudatus (ACA), Triticum vulgaris (WGA), Ulex Europaeus Agglutinin I (UEA-I), Phytolacca americana (PWM), Maackia Amurensis Lectin I (MAL-I), Galanthus nivalis (GNA), Bauhinia Purpurea Lectin (BPL), Phaseolus vulgaris Agglutinin (E+L) (PHA-E+L) and Sambucus Nigra Lectin (SNA) were purchased from vector laboratories, Sigma-Aldrich and Calbiochem (Merck), respectively. The detail information was showed in Table S1.

Lectin name	Specificity	Print monosaccharide	Supplied by
Jacalin	Galβ1-3GalNAcα-Ser/Thr(T), sialyl-T(ST)	Galactose	Vector
ECA	Galβ-1,4GlcNAc (type II), Galβ1-3GlcNAc (type I)	Galactose	Vector
HHL	High-Mannose, Manα1-3Man, Manα1-6Man, Man5-GlcNAc2-Asn	Mannose	Vector
WFA	Terminal GalNAc	GalNAc	Vector
GSL-II	GlcNAc and galactosylated N-glycans	GlcNAc	Vector
MAL-II	Siaα2-3Galβ1-4Glc(NAc)/Glc, Sia-Lex		Vector
PHA-E	Bisecting GlcNAc and biantennary N-glycans	GlcNAc	Vector
PTL-I	GalNAc, GalNAcα-1,3Gal, GalNAcα-1,3Galβ-1,3/4Glc	GalNAc	Vector
SJA	terminal in GalNAc and Gal	GalNAc	Vector
PNA	Galβ1-3GalNAcα-Ser/Thr(T)	Galactose	Vector
EEL	Galα1-3(Fucα1-2)Gal	Galactose	Vector
AAL	Fucα1-6GlcNAc(core fucose), Fucα1-3(Galβ1-4)GlcNAc	Fucose	Vector
LTL	Fucα1-2Galβ1-4GlcNAc, Fucα1-3(Galβ1-4)GlcNAc	Fucose	Vector
MPL	Galβ1-3GalNAc, GalNAc	GalNAc	Vector
LEL	(GlcNAc)n, high mannose-type N-glycans	LacNAc	Vector
GSL-I	αGalNAc,GalNAcα-Ser/Thr(Tn)and αGal	GalNAc	Vector
DBA	GalNAcα-Ser/Thr(Tn) and GalNAcα1-3Gal	GalNAc	Vector
LCA	Fucα-1,6GlcNAc(core fucose)	Mannose	Vector
STL	trimers and tetramers of GlcNAc, core (GlcNAc) of N-glycan, oligosaccharide containing GlcNAc and MurNAc	GlcNAc	Vector
PTL-II	Gal	Galactose	Vector
DSA	β-D-GlcNA, (GlcNAcβ1-4)n, Galβ1-4GlcNAc	GlcNAc	Vector
VVA	Terminal GalNAc and GalNAcα-Ser/Thr(Tn)	GalNAc	Vector
MAL-I	Galβ-1,4GlcNAc, Siaα2-3Gal, Galβ1-3GlcNAc, Siaα2-3	Galactose	Vector

GNA	High-Mannose, Manα1-3Man	Mannose	Vector
NPL	High-Mannose, Manα1-6Man	Mannose	Vector
ACA	Galβ1-3GalNAcα-Ser/Thr(T antigen)	Galactose	Vector
BPL	Galβ1-3GalNAc, Terminal GalNAc	Galactose	Vector
PHA-E+L	Bisecting GlcNAc and biantennary N-glycans and tetraantennary complex-type N-glycan	GlcNAc	Vector
SNA	Sia2-6Gal/GalNAc	GlcNAc	Vector
RCA120	β-Gal, Galβ-1,4GlcNAc(type II), Galβ1-3GlcNAc (type I)	Galactose	Sigma-Aldrich
BS-I	αgal and αGalNAc	Galactose	Sigma-Aldrich
PSA	α-D-Man, Fucα-1,6GlcNAc, α-D-Glc	Fucose	Sigma-Aldrich
SBA	Terminal GalNAc(especially GalNAcα1-3Gal)	GalNAc	Sigma-Aldrich
WGA	Mμltivalent Sia and (GlcNAc)n	GlcNAc	Sigma-Aldrich
UEA-I	Fucoseα1-2Galβ1-4Glc(NAc)	Fucose	Sigma-Aldrich
PWM	Branched (LacNAc)n	GlcNAc	Sigma-Aldrich
ConA	High-Mannose, Manα1-6(Manα 1-3)Man, terminal GlcNAc	Mannose	Calbiochem
