# Supplementary material for: Stage-associated differences in the serum N- and O-glycan profiles of patients with non-small cell lung cancer
Source: Clin Proteomics. 2019 May 10;16:20. doi: 10.1186/s12014-019-9240-6 (PMC6509814; doi:10.1186/s12014-019-9240-6)
Supplement: Supplementary file 3 — Additional file 3: Table S2. The normalized fluorescent intensities (NFIs) of each lectin for the serum proteins from NSCLC groups and control group. [file 12014_2019_9240_MOESM3_ESM.pdf]

Table S2. The normalized fluorescent intensities (NFIs) of each lectin for the serum proteins from NSCLC groups and control group

Lectin	Normalized fluorescence intensity (mean±SD)[a]						
	Adenocarcinoma (stage)			SCC (stage)			Healthy control
	I/II	III	IV	I/II	III	IV	
Jacalin	0.285±0.055	0.316±0.002	0.357±0.032	0.051±0.004	0.029±0.004	0.039±0.005	0.170±0.009
ECA	0.045±0.010	0.048±0.013	0.048±0.011	0.009±0.003	0.012±0.003	0.014±0.004	0.047±0.027
HHL	0.022±0.003	0.025±0.0002	0.025±0.001	0.020±0.002	0.018±0.001	0.020±0.001	0.008±0.004
WFA	0.047±0.002	0.043±0.002	0.043±0.003	0.022±0.003	0.016±0.002	0.021±0.001	0.030±0.001
GSL-II	0.014±0.002	0.012±0.0005	0.006±0.003	0.007±0.01	0.011±0.01	0.006±0.0099	0.001±0.005
MAL-II	0.006±0.002	0.005±0.001	0.005±0.002	—	—	—	0.004±0.001
PHA-E	0.026±0.0004	0.026±0.0005	0.026±0.001	0.040±0.002	0.045±0.005	0.043±0.003	0.033±0.003
PTL-I	0.015±0.002	0.017±0.002	0.012±0.002	0.029±0.003	0.029±0.001	0.028±0.002	0.004±0.0002
SJA	0.015±0.001	0.014±0.001	0.008±0.002	0.018±0.001	0.017±0.001	0.015±0.002	0.005±0.002
PNA	0.021±0.0002	0.024±0	0.020±0.003	0.035±0.007	0.025±0.013	0.042±0.005	0.039±0.005
EEL	0.017±0.002	0.019±0.001	0.016±0.003	0.007±0.006	0.001±0.0004	0.002±0.0006	0.011±0.001
AAL	0.007±0.0003	0.010±0.001	0.013±0.001	0.058±0.015	0.070±0.028	0.046±0.021	0.004±0.0002
LTL	—	—	—	—	—	—	0.007±0.001
MPL	0.025±0.002	0.028±0.012	0.026±0.003	0.011±0.018	0.005±0.009	0.018±0.016	0.027±0.015
LEL	0.014±0.003	0.020±0.001	0.020±0.002	—	0.010±0.017	0.018±0.016	0.032±0.001
GSL-I	0.006±0.0008	0.003±0.001	0.001±0.0001	—	—	—	—
DBA	0.006±0.0016	0.002±0.007	—	—	—	—	0.001±0.0003
LCA	0.015±0.002	0.010±0.001	0.006±0.001	0.039±0.026	0.021±0.024	0.032±0.024	0.009±0.0002
RCA-120	0.018±0.0001	0.014±0.002	0.012±0.001	0.061±0.008	0.064±0.009	0.055±0.005	0.141±0.008
STL	0.038±0.001	0.040±0.0005	0.039±0.001	0.031±0.001	0.028±0.008	0.029±0.006	0.088±0.005
BS-I	0.011±0.0001	0.009±0.0003	0.011±0.001	0.019±0.001	0.019±0.001	0.017±0.001	—
ConA	0.040±0.002	0.041±0.003	0.042±0.001	0.095±0.023	0.109±0.036	0.103±0.016	0.117±0.003
PTL-II	0.011±0.016	0.008±0.014	0.008±0.019	—	0.0005±0.0006	0.001±0.0006	—
DSA	0.028±0.0007	0.027±0.001	0.026±0.0003	0.030±0.001	0.022±0.014	0.028±0.0006	0.007±0.0004
SBA	0.021±0.0004	0.025±0.001	0.029±0.001	0.028±0.001	0.025±0.002	0.024±0.0003	0.002±0.000
VVA	0.021±0.0008	0.020±0.003	0.023±0.001	0.034±0.01	0.029±0.002	0.031±0.006	0.034±0.001
NPA	0.009±0.012	0.008±0.013	0.006±0.01	0.001±0.002	0.0005±0.0007	0.008±0.014	0.002±0.0003
PSA	0.055±0.008	0.035±0.001	0.036±0.004	0.069±0.059	0.096±0.078	0.082±0.047	0.006±0.0002
ACA	0.030±0.002	0.035±0.015	0.024±0.001	0.105±0.007	0.090±0.002	0.090±0.005	0.053±0.002
WGA	0.034±0.001	0.034±0.001	0.040±0.002	0.073±0.004	0.067±0.005	0.065±0.004	0.042±0.0007

UEA-I	0.006±0.003	0.004±0.0003	0.003±0.0004	0.006±0.001	0.006±0.0002	0.006±0.0001	0.001±0.0002
PWM	0.008±0.0003	0.006±0.0006	0.006±0.0006	0.012±0.002	0.011±0.001	0.010±0.001	0.011±0.0002
MAL-I	0.009±0.002	0.008±0.0006	0.005±0.0002	0.002±0.002	0.006±0.01	0.001±0.0003	—
GNA	0.013±0.001	0.013±0.0006	0.009±0.001	0.013±0.009	0.014±0.01	0.018±0.007	0.011±0.001
BPL	0.020±0.002	0.017±0.0003	0.014±0.004	0.027±0.001	0.026±0.001	0.021±0.003	—
PHA-E+L	0.010±0.003	0.011±0.002	0.010±0.004	0.030±0.001	0.040±0.003	0.034±0.004	0.003±0.0003
SNA	0.035±0.001	0.029±0.003	0.028±0.001	0.019±0.001	0.038±0.017	0.035±0.021	0.052±0.032

[a] The signals obtained for 9 repeated blocks in 3 repeated slides were averaged and its SD was counted. "—", negative signals.
